# Supplementary material for: Adaptive sport as affirmation: “We focus on our strengths, not our disabilities”
Source: PLoS One. 2023 May 10;18(5):e0283842. doi: 10.1371/journal.pone.0283842 (PMC10171584; doi:10.1371/journal.pone.0283842)
Supplement: Attachment — Submitted filename: Response to Reviewers.docx [file pone.0283842.s001.docx]

Yih-Kuen Jan, PhD

Academic Editor

PLOS ONE

February 10, 2022

Dear Dr. Yih-Kuen Jan:

This letter describes the changes we made in response to the points raised during the review process of our submission, PONE-D22-29815, Adaptive sport as affirmation: “We focus on our strengths, not our disabilities”.

Reviewer #1: “This manuscript is well written and presents a perspective about athletes with disabilities that is needed and rarely described. In a scientific field that often addresses pathology, this paper addresses the internal motivations and self-assessments of athletes who reject the limitations of the labels.”

Changes:

This reviewer did not require any changes.

Reviewer #2, Comment 1: “The reviewer applaud researchers for researching on this rare area. However, (a) Introduction should be short. Reviewer could not pick the difference between introduction and background to study. (b) The background to study should show the problem and its setting. researchers should revisit it and unpack a bit. Also, (c) research questions can help in focusing during data collection. With research questions, findings can be more focussed.”

Changes:

a. We substantially shortened the introduction, making a clearer separation from the background of the study.  P3.

b. We made significant changes to the background, addressing issues brought to our attention by readers of an earlier draft. Thus, we included more background on Victor Turner’s theories of liminality and communitas.Because people with impairments often feel like outsiders and newly disabled people have their lives suddenly turned around by trauma, we felt that these theories would be the most fitting. As one moves from the sense of being alone and excluded to that of being accepted by a community, they enter the new phase of *communitas*. P2

c. We added research questions. P3. Specifically, we added the following paragraph:

As part of a larger ethnographic project, the research questions aim to address the role of athletics in the lives of adaptive athletes. Specifically, we aim to understand the unique challenges, benefits and personal growth experiences that participants face in their chosen sport(s). What challenges, for example, does a newly disabled person face when entering or re-entering athletics?  How does participating in team sport alter one’s self-perception and view of life? For parents of children with impairments, how does being a part of an adaptive athletic community help their children grow and thrive? What is the range of emotions experienced by athletes and their families?

Reviewer 2, Comment 2: “Research findings are detailed and real as they were sourced verbatim from horse's mouth. However, whilst results are good, they tend to be too detailed on each participant. Most sound like biodata. More so, there is a tendency of over emphasising on certain participants, for example, Jesse Billauer Edwardo to mention a few. Bring out from them key issues relevant to your research questions. More so if these are not pseudor names, there is need to seek consent of publishing their names and their nature of disability so that their confidentiality is not violated.”

- We shortened the details on each participant without losing the context of their lived experiences. We spread out the emphasis to equalize the focus on each of the participants. Pp 15-26.
- We received consent to publish first names and photographs of a select group of participants. We used pseudonyms without photographs for the others.

Reviewer 2, Comment 3: “On another note, the discussion is an extension (sic) of findings. there is need to revisit it. Discussion should marry literature and findings such that conclusion is 'born' out of it. Thus there is need to revisit this area too.”

We rewrote the discussion section in order to bring it into better alignment with the literature and the findings, and to lead to the conclusion. In revisiting the discussion, we decided to include a more theoretical framework within the paper and discuss the work on liminality and *communitas* as theorized by anthropologist Victor Turner and fitting our ethnographic findings on adaptive athletics.

We have reviewed the reference list to ensure that it is complete and correct. We have not added or deleted any retracted references.

We appreciate your kind review of our manuscript and look forward to addressing any further issues that might need attention.

Sincerely,

Norah Anita Schwartz, Ph.D., MPA

Christine Alysse von Glascoe, Ph.D., M.D.
